# Microbial Musings: Winter 2023

**DOI:** 10.1099/mic.0.001331

**Published:** 2023-03-31

**Authors:** Andrew Preston

**Affiliations:** ^1^​ Department of Life Sciences, University of Bath, Bath, UK

## Abbreviations

AMR, antimicrobial resistance; OA, open access; RND, resistance-nodulation-division.

## Full-Text

This is the first edition of *Microbial Musings* since *Microbiology* moved to a fully open access (OA) format, so this may be the first time you are reading *Musings*. Here, editors highlight particular publications from recent issues. The move to being fully OA is in keeping with the direction of science publishing more widely. However, cost to authors is viewed as a major issue with OA publishing. The Microbiology Society offers a Publish and Read agreement for institutions or groups of institutions that enables authors to publish papers in the suite of Society journals without OA charges. Further details are available here. Publishing in Society journals helps to support our own research communities; the Microbiology Society feeds proceeds from publishing into supporting its members, such as through travel grants that support the attendance of students and other researchers at conferences and meetings, and supporting Annual Conference and other focused meetings, not all of which are in the UK.

A standout theme of recent *Microbiology* publications has been the role of efflux systems in antimicrobial resistance (AMR). Bacteria possess multiple different efflux systems, some of which are substrate specific while others are promiscuous. Of the numerous different types of efflux systems, the resistance–nodulation–division (RND) family is perhaps the best characterized and is recognized as being responsible for efflux of a wide range of antibiotics [1]. Efflux protects cells from a wide range of otherwise inhibitory compounds, some of which are exogenous while others are by-products of cellular metabolism. Efflux is intricately entwined with cellular physiology and numerous activators and inhibitors of efflux are known [2]. While not a new observation, the increasing number of studies that identify efflux as a key player in AMR suggests that circumventing this ability of bacteria to reduce the effective concentration of antimicrobials at their target sites is essential for overcoming the AMR pandemic.

*Pseudomonas aeruginosa* is an antibiotic-resistant public health threat in its own right, but has also served as a model for the study of the evolution of resistance [3]. The role of biofilms in enhancing the antibiotic resistance of bacteria, including *P. aeruginosa *, is established [4]. Interestingly, the resistance of *P. aeruginosa* to meropenem in dual-species biofilms comprising the bacteria and *Candida albicans* is greater than that of *P. aeruginosa* in a single-species biofilm [5]. The more robust biofilm built by the two species is thought to provide greater physical protection to *P. aeruginosa* from antibiotics.

Alam *et al*. sought to identify the responses of *P. aeruginosa* to the presence of the fungi that might also contribute to the increased resistance [6]. The authors performed transcriptional profiling to screen for differences in *P. aeruginosa* gene expression between single- and dual-species biofilms. For both single- and dual-species biofilm *P. aeruginosa,* meropenem induced a significant increase in transcription of the beta-lactamase-encoding *ampC*, a well-known determinant of meropenem resistance. The response also included increased expression of genes involved in biofilm formation, indicating the role of the biofilm niche in protection against exogenous insults. The presence of *Candida* had a surprisingly modest effect, but did promote the expression of several efflux pumps in *P. aeruginosa *, and the authors hypothesize that this contributes to the increased meropenem resistance observed for the dual-species biofilm bacteria. It could be that the increased expression of efflux pumps is aimed at protecting the bacteria from fungal-derived inhibitors, while also conferring increased resistance to other compounds — results that highlight the importance of efflux for protection of bacteria against antimicrobials.

In the same edition, Bové *et al*. also identified efflux as being key to the decreased antibiotic susceptibility of *P. aeruginosa *, this time of strains evolved in the presence of tobramycin and the inhibitor of quorum sensing, furanone C-30, that displayed reduced susceptibility to tobramycin [7]. These evolved strains carried mutations in *mexT* (encoding a transcriptional activator) and *fusA1* (elongation factor G, in which mutations protect from aminoglycoside antibiotics) and displayed altered growth rate and metabolic activity (measured by calorimetry) from wild-type (WT), indicating that the evolved phenotypes carried a fitness cost. Intriguingly, the virulence of the evolved strains in two different models was unchanged from WT, contrary to the idea of a trade-off between resistance and fitness in the absence of antimicrobial selection. A key component of the increased resistance of these strains was higher levels of expression of *mexE*, a component of the MexET-OprN efflux pump. *MexE* upregulation conferred resistance not only to the tobramycin, the selection to which the strains were evolved, but other classes of antibiotics too.

Moving away from *Pseudomonas,* Holden *et al*. conducted genome-wide mutagenesis, using TraDIS-*Xpress,* in *Escherichia coli* and *Salmonella typhimurium,* to catalogue genes involved in efflux of acriflavine, considered a canonical substrate of the AcrAB RND-type efflux system [8]. Clearly, toxic compounds can induce pleotropic effects in cells, so in this study the TraDIS screen was performed in the presence of acriflavine, and also in the presence and absence of the efflux inhibitor phenylalanine-arginine β-naphthylamide, enabling identification of genes involved in expression of the efflux system rather than other drug resistance effects. Through careful use of subinhibitory and inhibitory concentrations of acriflavine and PAβN, 37 *E. coli* and 34 *S *. *typhimurium* genes were identified as being involved in expression of acriflavine efflux. In addition to identification of the Acr efflux system, established regulators of *acrAB* expression were identified in the screen, validating the approach. Other transport systems were involved in efflux, as were glutathione metabolism, systems involved in cell membrane maintenance, and general stress responses. Several of these were suggested to act via a direct effect on regulators of the Acr system, highlighting that efflux is a central component of the response to a wide range of stresses and toxic compounds. On the whole, the same systems were identified in *E. coli* and *S. typhimurium,* suggesting that the regulation of efflux as a generalized stress response is an evolutionarily conserved feature. The study identifies numerous cellular systems important for the expression of efflux. The need to circumvent efflux for efficacious antimicrobial treatment suggests that targeting these systems could potentiate the activity of antibiotics, increasing their efficacy and longevity, identifying potential new targets for new classes of combination inhibitors.

Inhibition of efflux itself provides an attractive target for the development of novel compounds that will potentiate the effects of existing and future antibiotics [9]. However, the complexity of the key efflux systems provides a major challenge for efflux-blocking drug design. The promiscuity of many efflux systems for substrates creates complexity and difficulty for e.g. interpreting the structural effects of point mutations. A major question has been, how can a single system have substrates that vary so widely in their physico-chemical properties? For example, *E. coli* AcrB can transport macrolides, fluoroquinolones, tetracylines, acriflavine and other seemingly structurally disparate compounds [10]. Computational studies have greatly benefited the understanding of structures and mechanisms of numerous different biological systems. They have been applied to bacterial efflux systems, often with the aim of assisting in drug design.

Athar and colleagues from the University of Cagliari ask, what have we learned from computational studies of the RND superfamily of efflux pumps? [11]. This review compares studies that have attempted to characterize the binding sites for different efflux substrates. Unlike the classical picture of substrate binding, in which the substrate fits with high specificity into a binding pocket, the RND efflux proteins contain two major substrate binding regions, the access pocket (AP) and the distal pocket (DP). Remarkably, different substrates are bound using different types of interactions, and seemingly across different regions of these sites, explaining the polyspecificity of the efflux systems. Efficient efflux of substrates requires them to bind within these pockets with sufficient strength to be retained within them, but while still allowing the movement of the substrate within the structure in response to conformational changes in the efflux proteins, as efflux requires the substrate to move into the membrane fusion protein that spans the periplasmic space, linking the inner and outer membrane components.

Computer simulations have been key to modelling the proposed conformational changes involved in efflux, and the cyclical movement of substrates between the different components of the systems, including understanding the complex energetics of the system. In addition to utility in understanding the binding of the varied natural substrates of efflux systems, computational simulations of the binding of known inhibitors promises to help us understand the basis for inhibition as opposed to effective efflux — critical when considering efflux as a target for drug design. The authors highlight the need to identify features conserved among different efflux systems. Many bacteria possess multiple efflux systems. As each can efflux multiple different compounds, inhibitors that target a specific system are likely to be of limited use in combatting efflux of antibiotics. Computational approaches are well suited to identifying features common to different efflux systems, and thus guiding broad-spectrum efflux inhibitors. The dramatic increase in computing power over recent years has increased the complexity of computer simulations and the authors recognize the importance of including the highly complex and variable environments of the bacterial membranes in which efflux systems sit as key determinants of the mechanism by which efflux pumps recognize different substrates and activate efflux.

Finally, the involvement of efflux in AMR is not limited to bacteria. Islam and colleagues discuss the situation in fungi and pose the intriguing question, do mitochondria use efflux pumps to protect their ribosomes from antibiotics? [12]. Efflux is established as a key mechanism by which fungi limit the concentration of antibiotics at their target sites, with efflux out of the cell or into vacuoles. Fungi exist in many of the same highly competitive niches that have driven the antibiotic/resistance arms race that is well discussed for bacteria, and thus it should be no surprise that fungi have evolved under the selective pressure of antifungal compounds. Mitochondrial ribosomes are susceptible to a number of antibiotics that target bacterial ribosomes. However, protein translation of isolated fungal ribosomes is inhibited by much lower levels of these antibiotics than is required to inhibit translation in intact mitochondria, suggesting that the mitochondria provide protection from the antibiotics. Ethidium bromide efflux assays demonstrate the activity of mitochondrial efflux pumps, raising the possibility that efflux is a common mechanism of antibiotic resistance for both bacteria and mitochondria. The authors survey fungal genomes for genes encoding efflux systems and reveal that they contain a myriad of such systems, but the cellular location of many is unclear. The authors conclude that this knowledge gap means the answer to their original question is, at present, largely unknown.

The world is slowly recovering from the coronavirus disease 2019 (COVID-19) pandemic. As we enter a period of reviewing the lessons learned, it is clear that being better prepared for future infectious disease threats must be one of the main outcomes. Microbiologists would argue that while antibiotic resistance is often discussed as a looming threat, it is already very much a clear and present danger. While the number of publications in the area of AMR is great, they demonstrate that the danger of AMR is well recognized, but solutions to the problem are still some way off.

However, the emergence from the immediate shadow of COVID-19 does at least mean the Microbiology Society Annual Conference in Birmingham this month (17–20 April 2023) will once again provide the forum for us to meet with colleagues from around the world, and just maybe, have those in-person conversations that could spark a project to provide one of the solutions.

## References

[1] Li XZ, Plésiat P, Nikaido H (2015). The challenge of efflux-mediated antibiotic resistance in Gram-negative bacteria. Clin Microbiol Rev.

[2] Colclough AL, Alav I, Whittle EE, Pugh HL, Darby EM (2020). RND efflux pumps in Gram-negative bacteria; regulation, structure and role in antibiotic resistance. Future Microbiol.

[3] Diggle SP, Whiteley M (2020). Microbe profile: *Pseudomonas aeruginosa*: opportunistic pathogen and lab rat. Microbiology.

[4] Mah TF, O’Toole GA (2001). Mechanisms of biofilm resistance to antimicrobial agents. Trends Microbiol.

[5] Alam F, Catlow D, Di Maio A, Blair JMA, Hall RA (2020). *Candida albicans* enhances meropenem tolerance of *Pseudomonas aeruginosa* in a dual-species biofilm. J Antimicrob Chemother.

[6] Alam F, Blair JMA, Hall RA (2023). Transcriptional profiling *of Pseudomonas aeruginosa* mature single- and dual-species biofilms in response to meropenem. Microbiology.

[7] Bové M, Kolpen M, Lichtenberg M, Bjarnsholt T, Coenye T (2023). Adaptation of *Pseudomonas aeruginosa* biofilms to tobramycin and the quorum sensing inhibitor C-30 during experimental evolution requires multiple genotypic and phenotypic changes. Microbiology.

[8] Holden ER, Yasir M, Turner AK, Wain J, Charles IG (2023). Genome-wide analysis of genes involved in efflux function and regulation within *Escherichia coli* and *Salmonella enterica* serovar Typhimurium. Microbiology.

[9] Wang Y, Venter H, Ma S (2016). Efflux pump inhibitors: a novel approach to combat efflux-mediated drug resistance in bacteria. Curr Drug Targets.

[10] Kobylka J, Kuth MS, Müller RT, Geertsma ER, Pos KM (2020). AcrB: a mean, keen, drug efflux machine. Ann N Y Acad Sci.

[11] Athar M, Gervasoni S, Catte A, Basciu A, Malloci G (2023). Tripartite efflux pumps of the RND superfamily: what did we learn from computational studies?. Microbiology.

[12] Islam MD, Harrison BD, Li JJ-Y, McLoughlin AG, Court DA (2023). Do mitochondria use efflux pumps to protect their ribosomes from antibiotics?. Microbiology.

